# Collectanea

**Published:** 1827-10

**Authors:** 


### COLLECTANEA.

Floriferis ut apes in saltibus omnia libant,
Omnia nos, itidem, depascimur aurea dicta.

### ANATOMY.

*Unusual Variety in the Femoral Artery.*—The following is described by Mr. J. Houston, in the *Dublin Hospital Reports.*

The femoral artery, at its commencement, was of the ordinary size, and in the place where it is always found. About one inch and a quarter below Poupart's ligament, it sent off the profunda, which at first descended behind it, and not as usual along its outer side. This branch formed a short trunk which passed inwards, and soon furnished the circumflex and perforating arteries, with several smaller twigs that went to the groin and its neighbourhood.

About half an inch from the origin of the profunda,—viz. one inch and three-quarters below Poupart's ligament, the femoral artery divided into two branches, an internal and an external, the former of which was somewhat the larger; but though either of them, taken singly, was less than an ordinary-sized femoral, yet both combined would have made a vessel of larger calibre. These branches lay together on the same plane, and in such order descended parallel, as far as the opening for them at the lower part of the thigh in the tendon of the triceps, at which point precisely they reunited to form the popliteal artery.

These vessels bore the same anatomical relations to the parts in their vicinity, and followed exactly the same course as the femoral, which they represented: each, however, was enveloped in a distinct fibrous sheath, which kept them so apart that an incision exposing but one of them would not have brought the other into view, though the only space between them was that occupied by the intervening layer of fascia.

The branches furnished by these vessels were four in number,—three from the external and one from the internal. Of the external three, the first, about as large as a crow-quill, came off close to the division, and went backwards to the muscles behind it; the second was smaller, and entered the vastus internus; and the third, which occupied the place of the anastomotica magna, took its origin just as the artery was joining its fellow. The branch from the internal vessel was small, and lost itself in the adductor muscles.

The popliteal, formed by the reunion of the two femoral arteries, was of the usual size; and, either in its distribution or anatomical relations, offered no one peculiarity.

The femoral vein, unlike the artery, was single. Placed superiorly along its inner side, it gradually, in passing the middle of the thigh, got to its back part, and applied itself in the ham to the outside of the popliteal artery, preserving throughout its entire course more relation to the internal than to the external arterial trunk.

The crural nerve presented nothing unusual. A few small twigs passed from it over the vessels in the upper third of the thigh, and lower down the saphenus branch attached itself to their sheath for some way, and thence proceeded towards the internal condyle of the femur.

Every circumstance connected with the irregularity in this case was such as must have inevitably caused a failure, had the occurrence of an aneurism in the ham unluckily made the operation necessary. The bifurcation of the femoral artery above the place at which it is usually tied, with the reunion of the branches in the lower part of the thigh; the concealment of the profunda, and the occupation of its place by the external division; together with the double sheath, was a combination incompatible with success. The surgeon would, most likely, have tied his ligature round the internal division of the artery, with perhaps only a passing observation on its smallness; whilst the external, either being unnoticed on account of its fibrous covering, or else mistaken for the profunda, and consequently left unobstructed, would have carried the blood with sufficient force to make the tumor pulsate, and frustrate the design of the operation. The same result must have equally followed had the external division happened to become the subject of the surgeon's selection.

The preparation showing this variety I have preserved, and placed in the Museum of the Royal College of Surgeons.

[This case is precisely analogous to the one published by Mr. CHARLES BELL, in the Number of this Journal for August 1826.]

---

*Description of a Human Stomach, of a singular Form and Structure.*—In the course of the last winter, (says Mr. HART,) I had an opportunity of examining a human stomach of so singular a form, that it is scarcely necessary to apologise for troubling the profession with an account of it. The body in which it occurred was that of a female, aged about thirty, which had been brought into the Park-street dissecting-room; nothing of whose previous history could be learned.

An intelligent pupil, to whom the body had been allotted for dissection, first called my attention to it by remarking that the abdominal viscera appeared to be somewhat displaced. On making examination, I found that the stomach deviated from its usual transverse direction, approaching more to the perpendicular; and that the first portion of the duodenum appeared unusually long, running transversely to the right without ascending, as generally described, in its course from the pylorus to the neck of the gall-bladder.

As the irregular position of these organs might have been a consequence of disease, I examined with particular care the state of the surrounding viscera, more especially the condition of the peritoneum, but no morbid appearance whatever, not even the existence of a single adhesion of the last-named organ, could be discovered.

The stomach and duodenum were then removed and inflated, when the peculiar shape of the former viscus, and its remarkable connexion with the latter, became fully apparent. The left extremity of the stomach had its usual appearance as to form and size; but the right extremity, instead of contracting until it became continuous with the duodenum after the ordinary way, ended in a cul-de-sac, about half as large, to appearance, as the great end of the left extremity. The duodenum proceeded from a depression which marked the lesser arch of the stomach, about midway between its cardiac orifice and the most remote part of its right extremity.

When I injected air, and afterwards water, into the stomach, by the cardiac orifice, I found that these fluids were longer than usual in finding their way into the duodenum, not entering this latter viscus until after the stomach had become fully distended; a circumstance which was not owing to any preternatural contraction of the pylorus, but depended altogether on the acuteness of the angle at which the duodenum went off from the stomach.

The stomach exhibited a very remarkable appearance in the vicinity of the pyloric orifice, in consequence of a patch of tendinous substance on the anterior, and another on the posterior surface of that organ, each of which was of a form nearly circular, and about twice the diameter of a crown-piece.

The circular fibres of the muscular coat were arranged thus at the right extremity of the stomach; they ran converging from the circumference of the cul-de-sac to insert themselves into the margin of the tendinous patches above described. The longitudinal fibres of the duodenum arose from the opposite edges of these tendons, which thus served as pyloric ligaments.

On examining the pylorus, by inverting the duodenum, I found that it consisted of a slit-like opening, having a transverse direction with respect to the line of the lesser arch of the stomach; and that it was formed between two semicircular folds of the mucous membrane, including muscular fibres, resembling very much the ileo-cœlic valve. These muscular fibres, which entered into the structure of the valve, were the commencement of the circular fibres of the duodenum. The biliary and pancreatic ducts opened into the lower part of the descending portion of the duodenum in the usual way.

The peculiar form of the stomach here described, the presence of the patches of tendon on its surfaces, and the converging arrangement of the muscular fibres at the right extremity, gave it an appearance strongly resembling that form of stomach which exists in several tribes of predaceous birds. In fact, it had quite the appearance of a stomach which an anatomist would feel disposed to say had belonged to a carnivorous animal.

It might, perhaps, have been interesting to ascertain whether there had existed any peculiarity in the digestive function of the individual to whom this stomach belonged; but there was no information on this point which could be obtained, as I could not succeed in learning any thing of her history during her lifetime. (*Ibid.*)

Cruelty to animals

### PHYSIOLOGY.

*Account of Experiments with three Species of Indian Serpents, with a view to ascertain the comparative Virulence of their Poison.*—These experiments were made by Mr. Breton, and are related in the second volume of the *Transactions of the Medical and Physical Society of Calcutta.*]

On the 19th of April, 1825, a Cobra de Capello having been brought to me, it was made to bite the thigh of a pigeon, and in three minutes it expired.

April 20.—In the presence of W. Twining, Esq. surgeon to his Excellency the Commander in Chief, a rabbit was bitten, and it died in three minutes.

Immediately after, a pigeon was bitten, and in four minutes it was dead.

A second pigeon was then bitten, and it survived only eight minutes.

After an elapse of about twenty minutes, a third pigeon was bitten, and it was lifeless in three minutes. In this instance there was probably a fresh secretion of the poison, which was the cause of its activity.

A fourth pigeon was then bitten, and about a quarter of an hour afterwards blood and lymph, resembling sanies, oozed from the wounds inflicted by the fangs. The pigeon immediately couched, and remained on the ground without moving, gradually drooped, and in fifty-eight minutes expired.

A fifth pigeon was bitten, immediately after the fourth pigeon was disengaged from the fangs of the snake, and it survived four hours and twenty minutes.

The rabbit and pigeons were bitten by putting the thigh of each to the mouth of the snake, while the latter was held by the throat by the snake-catcher. On diminishing the compression on the snake's throat, the reptile readily seized the part presented to it, and bit it forcibly, retaining its hold till the animals and snake were thrown together on the ground. The very instant the pigeon on the 19th, and the rabbit and four pigeons on the 20th, were bitten in the thigh, the limb became paralysed, apparently from the effects of the poison, and not simply from the infliction of the wounds; for the last pigeon that was bitten, (the wounds in the thigh being very apparent,) manifested not the least affection of the limb until within an hour of its death, when muscular motion, first of the legs, and then of the body, gradually declined till the pigeon expired, evincing the weakness of the poison of the snake after a succession of emissions of it.

For about three hours, the pigeon last bitten did not appear at all affected by the poison. It then began to manifest restlessness, by fluttering now and then in the basket in which it was placed, and by throwing its head and neck in every direction, and gasping for breath, until it expired. Muscular motion seemed to cease first in the legs, next in the body, then in the wings, and lastly in the neck and throat.

The symptoms manifested in the other pigeons and rabbit were instantaneous paralysis of the bitten limb, torpor, and slight convulsions. In neither of these animals, kept for several hours after death, was there apparent more than ordinary tendency to putrefaction.

On the 23d of April, 1825, at twenty-three minutes after ten A.M., in the presence of Assistant-surgeons Egerton and Macfarlane, a full-grown young dog was bitten by a Cobra de Capello in the thigh, and he instantly expressed pain, and limped a little. The wound appeared to remain painful, for he gently licked it several times. For upwards of an hour, no general effect of the poison was apparent. The dog then began to manifest restlessness and languor, lying down, and rising at short intervals, with but little power to support himself in a standing posture. He gradually drooped; was latterly slightly convulsed; his tongue a few times ejected from the mouth, and with difficulty retracted; had a little foaming and discharge of saliva; a few successions of tremors, of short duration, of the body; and he expired at the expiration of two hours and thirty-one minutes after being bitten. The dog had no vomiting; the bitten limb was slightly swollen; and the body, although kept several hours, presented no other than ordinary tendency to putrefaction.

The slow action of the poison on this dog may possibly be ascribable to the snake having been, three days before, made to bite a rabbit and five pigeons, which may have in some degree weakened the action of the poisonous secretory organs.

At twenty-five minutes after ten A.M. a second dog, three parts grown, was bitten in the thigh. He instantly manifested pain, and limped a little, but remained the whole day lively and apparently well, and at night eat as usual his food. The following morning, the whole of the bitten limb was swollen and paralysed. At about nine A.M. languor was apparent, which continued till the evening, when he revived, and gradually recovered from the effects of the poison, and of the paralysis of the limb.

During several hours, the evident symptoms were torpor; very slow and difficult respiration; languid circulation; a little foaming and discharge of saliva; a convulsive ejection from the mouth a few times of the tongue, with difficulty of retracting it; dilatation of the iris; and now and then a tremulous motion of the body.

In this experiment a fact is established, that, although the effect of the venom of a serpent may be for several hours very evident, an animal is capable, without any remedy whatever, of surviving its action; for, the day after being bitten, the dog remained several hours apparently in a dying state, but in the course of the following day recovered perfectly.

The third and fourth, half-grown whelps, were bitten in quick succession to the second dog; and, although they both manifested pain, and limped after being bitten, no effect of the poison occurred.

These experiments prove that, after the first or second emission of the poison, it becomes too weak to destroy even a whelp three-parts grown.

Immediately after the fourth dog, a pigeon was bitten in the thigh by the same snake. The limb was instantly paralysed, and it gradually drooped, and died in twenty-one minutes.

From the above experiments, it would appear that the venom, although not sufficiently powerful to kill either of the three whelps, was yet active enough to destroy a small animal, as a pigeon, in twenty-one minutes.

*Experiments with a second Cobra de Capello.*—An innoxious water-snake, called Dhonr, was bitten towards the tail. For an hour and a half, it showed no sign of being affected by the poison. It afterwards lost the use of the portion below the bitten part, gradually became languid, and, without any other symptom than gasping for breath, died in two hours and fifteen minutes.

2d. A rabbit, bitten in the thigh immediately after the water-snake, had its limb instantly paralysed, and, with no other symptom than torpor and slight convulsion, died in eleven minutes. The part bitten became black, and there was a little extravasation of dark-coloured blood around the bitten part.

3d. Immediately after the rabbit, a pigeon was bitten, and it died in twenty-seven minutes.

4th. The second pigeon, bitten in immediate succession, died in one hour and eleven minutes.

5th. The third pigeon bitten drooped, and died in three hours and forty-two minutes.

6th. The fourth pigeon bitten showed no symptom of the poison.

7th. The fifth pigeon bitten was not at all affected.

The water-snake, rabbit, and five pigeons, were bitten in immediate succession, while the Cobra de Capello was held by the neck by the snake-catcher. And these experiments prove—1st. That an innoxious snake can be killed by the venom of a poisonous serpent. 2d. That the second emission of the poison is speedily fatal to a small animal, as the rabbit. 3d. That the poison becomes gradually weaker after every successive emission of the poison, until it becomes inert; or possibly, after a certain period, there is for a time a cessation of the secretion of the poison, for the two pigeons last bitten remain unaffected.

Rabbits and pigeons appear to be equally speedily killed by the venom of serpents. They generally die in two or three minutes after being bitten by a Cobra de Capello. Full-grown dogs are killed by the venom of this snake in full vigour in fifteen or twenty minutes.

On the 30th of April, the snake-catchers brought me a venomous snake, called by them Bora. On examination, it was identified with the Kutuka Rekula Poda of Dr. P. Russell, which in Behar is known by the name of Amaiter and Seeah Chunder. In the upper jaw of this snake there were four poisonous fangs, two on each side; and as they were, with their muscular sheaths, large and very distinct, they were exhibited to the medical students, and to others who were present.

The fangs of this snake were by the snake-catcher forced into the thigh of a whelp three-parts grown. He immediately limped, but for upwards of an hour manifested no other effect of the poison. He then seemed restless, breathed with difficulty, gradually lost the use of his hind legs; and, without any vomiting, and with but slight convulsion, drooped and died in four hours and eight minutes.

Immediately after this whelp was bitten, another half-grown whelp was wounded in a similar manner by the fangs of this snake, but without any effect of the poison being evinced.

In the same manner, and in immediate succession, a third whelp and a pigeon were punctured in the thigh by the snake's fangs, but no effect was produced in either of these animals.

In forcing the fangs into the thighs of the animals, one of the fangs was torn off from its sheath, and it fell on the ground. I had it picked up, and it is now in my possession, with a horse-hair passed through the hollow of it.

After the last experiment, the Bora was bitten by a vigorous Cobra de Capello, but no effect whatever of the poison appeared.

Immediately after, and by the same Cobra de Capello, a pigeon was bitten in the thigh, and it died in sixteen minutes; a proof of the activity of the poison, even after the second emission of it.

May 1st.—The Bora and Cobra de Capello were irritated, and then brought together, and made to bite each other a few times. No impression of the teeth of either of the snakes being visible, a few of the scales on the belly of both the snakes were scraped off, and the fangs of each forcibly introduced, and retained a few seconds in the wounds. The punctures were distinct in the belly of both the snakes, but neither of them were affected by the poison.

From this experiment it would seem that a poisonous snake is unsusceptible of the poison of another species of snake.

May 3d.—The same Cobra de Capello, in full vigour, bit a whelp in the nose, and it died in sixteen minutes.

An attempt was made to make the Bora bite a whelp; but, in the act of biting, two of the three fangs remaining in the Bora's upper jaw being torn off from their sheaths, fell on the ground. These are preserved, and a horse-hair passed through them, to show they are poisonous fangs. The Bora, being unable to renew its bites, was put in a bottle of spirit of wine.

On the 3d of June, the snake-catchers brought me for experiment a third kind of venomous snake, named in Russell's work on Indian Serpents, Bungarum Pamah, and in Shaw's Zoology, Boa Fasciata, and called in Bengal Saunkenee. Its poison proved less virulent than that of the Cobra de Capello and Katuka Rekula Poda; for, of four pigeons bitten at intervals of a few minutes, the first died in nineteen minutes; the second in twenty minutes; the third in fifty-two minutes; and the fourth in forty-six minutes. Two of the pigeons were opened immediately after death, and the heart and large vessels examined: the blood was not in any part coagulated, and none was contained in the ventricles of the heart.

---

*Account of the Death of Mr. Drake by the Bite of a Rattle-snake.*—At the meeting of the Academy of Sciences of France on the 9th April last, some documents were presented by M. Dumeril, connected with the death of Mr. Drake by the bite of a rattle-snake, forming part of a collection of reptiles which that person had exhibited at London, and had taken to France for the same purpose. These documents were transmitted to the Academy by the minister of the interior; and seem to have excited fears in some of the members, lest, the climate of France being favourable, some of these dangerous reptiles might escape and propagate.

From these documents it appears that Mr. Drake arrived at an inn in Rouen on the 8th February, with three live rattle-snakes and some young crocodiles, and that, notwithstanding his care to preserve them from cold on the road, he saw with grief, on his arrival, that the finest of the three was dead. The dead animal was removed from the cage, and the cage itself, with the other two, were taken into the dining-room, and placed near the stove. Here Mr. Drake endeavoured to rouse them with a stick; but, perceiving that one of the two gave no signs of animation, he opened the cage, took the serpent by the head and tail, and approaching a window to ascertain by handling if life was extinct, the animal turned its head half round, and fixed one of its fangs in the posterior external part of the left hand. Mr. Drake shrieked, pronounced some words in English, according to the reports, and was replacing the serpent in the cage, when it again bit him on the palm of the same hand. Mr. Drake now run out into the court, calling eagerly for a surgeon; and, not finding water readily, rubbed his hand upon the ice, which he found at the door. Two minutes after, having procured a cord, he himself made a ligature on the arm above the hand. Notwithstanding these precautions, his agitation from the fear of the consequences continued to increase till the arrival of Dr. Pihorel. The presence of this gentleman somewhat composed the feelings of Mr. Drake; and he saw with eager joy the chafing-dish and irons arrive, with which the wounds were to be cauterised. This operation was instantly performed, and the patient took internally half a glassful of olive-oil. Drake seemed now to have resumed his tranquillity; but, in a few minutes more, symptoms made their appearance which rendered the case hopeless, and he died in eight hours and three-quarters after the bites.

The body was afterwards opened. The internal organs appeared healthy; the brain and spinal cord were unaltered. The membrane which covered these parts, however, was observed to have a reddish tinge. The veins presented no trace of inflammation; and the only appearance of derangement in the system consisted in the veins of the affected side having the blood curdled or clotted.

The physicians of Rouen, where the accident happened, and who examined the body, recommended that, in future, exhibitors of dangerous serpents should be obliged to extract the poison-fangs, and be constantly provided with cupping-glasses and instruments for cauterisation; and the commission of the Academy coincide in that opinion. But it was remarked, that the successive growth of these teeth would require them to be removed every two or three months, or as soon as they were reproduced. The commission recommended suction of the wound as one of the most efficacious measures for the extraction of poison; and state that this, even when done by the mouth, is attended with no danger to the operator, provided the mouth which sucks be sound. The ligature of a place above the wound, imperfectly done in Mr. Drake's case, was strongly recommended by Dr. Magendie.

The melancholy termination of this case induced many of the members of the Academy to propose the absolute interdiction of the exhibition of poisonous animals for the gratification of public curiosity. M. Geoffroy, to demonstrate the virulence of the poison of the rattle-snake, mentioned that the body of the reptile which had bitten Mr. Drake had been sent to the Museum of Natural History; and that, eight days after its dissection, one of the assistants having punctured his hand with the scalpel employed in this operation, the slight wound was followed by nearly serious consequences. The hand swelled, and severe pain in the axilla supervened; symptoms similar to what occur in this country from scratches and punctures received in dissection, without any reference to a specific poison.

M. Dumeril remarked, that the effects of the bite of the rattle-snake in America was much less sudden and less fatal than in the case which had unfortunately happened at Rouen; and M. Bosc stated, that, of all venomous animals, the rattle-snake was the most gentle, and never attempted to bite where flight was possible. He had seen, he said, more than thirty persons bitten by rattle-snakes, none of whom died. A horse, however, which had been bitten on the tongue, fell a victim to the poison.

The discrepancy in the result of the cases may, perhaps, be accounted for, by supposing that the virulence of the poison in the rattle-snake may not be the same at all periods of the year, or of the animal's life. In some cases, however, and Mr. Drake's is one of them, the poison seems to act speedily and fatally. In a curious Memoir on the Habits of the Rattle-snake, read lately by Mr. Audubon at the Wernerian Society, that gentleman mentioned a circumstance which tends to show that the poisonous fangs of this reptile, even when withdrawn from the animal, retain their virulence for years. A person had been bitten by a rattle-snake in the woods through a strong boot. He died without the cause of his death being properly investigated. The boots descended to his son, who, after putting them on, was suddenly taken ill, and also died. The effects of this last were brought to sale; and a younger brother fancying the boots, or willing to preserve some memorial of his father and brother, was the purchaser. He used them only once, when he also fell ill, and died. The medical men, whom such an occurrence had led to investigate its cause, at last ripped up the fatal boot, and found, firmly fixed in the substance of the leather, the fang of the rattle-snake, which had thus caused the death of three individuals. American Farmer.

Rattle-snakes, Mr. Audubon further observed, are often found coiled up and torpid when the temperature is low; and he himself once narrowly escaped from perhaps a serious accident, in trusting to their continued torpidity. He had found an excellent specimen coiled up and torpid, which he put in his knapsack along with some wild ducks he had been shooting. The motion and heat of his body, together with the additional heat afforded by a sportsman's fire at a meal in the woods, had however revived the animal; and the motions of his knapsack, observed from the outside, indicated life within. Mr. Audubon at first thought that some of his ducks, imperfectly killed, had found their situation irksome, and were testifying their impatience; but the recollection of the rattlesnake flashing at once on his mind, he threw off his bag, ducks, reptile, and altogether. The removal of the animal to a colder temperature brought on again its torpidity. He carried the snake home; and the identical specimen, if we rightly understood him, is now in the Museum of the Lyceum of Natural History of New York. (*Edinburgh Journal of Science.*)

---

### PATHOLOGY.

*Theory of Fever.*—The following is from the pen of Dr. Bow, of Alnwick.

As the nature of the proximate cause of fever is still disputed, I think that any effort, however humble, to unravel the mystery, cannot but be acceptable to the readers of your useful Journal.

In ague, and in fevers arising from mental emotions, cold, &c. the first effect of the remote cause I conceive to be simply impaired energy of the nervous system; owing to which, the products of secretion throughout the body become imperfect in their chemical constitution. This imperfection results from the want of the force of nervous energy necessary to determine the proper decomposition of the fluid acted on, and the proper recombination. Sooner or later, these imperfect products irritate (as would foreign matter) the nervous extremities with which they are in contact, and this irritation is the cause of the reaction—is the cause of the hot stage. I believe it will in general be found that, in all fevers, sensations of heat on the surface precede increased action of the sanguiferous system; and therefore I am led to conclude that increased action of the sanguiferous system is not the cause of the heat. The inordinate action of the heart results from the absorption of the imperfect products of secretion. As soon as the brain and spinal marrow are roused by the irritation produced by the vitiated secretions, energy is transmitted in force sufficient to effect healthy secretion, and the absorbed imperfect products are soon thrown off by the skin or otherwise. But it is not so with contagious fevers: here I suspect that, besides impaired energy, there is functional disorder of the brain and spinal marrow; hence nervous energy will not only be deficient in force, but will also be imperfect in its nature. If so, the explanation is easy why these fevers should be continued. Although, as in ague, the brain and spinal marrow be roused by the irritation produced, yet the energy transmitted is imperfect, and therefore unfitted for the purpose of secretion: hence secretion will continue to be imperfectly performed;—hence will continue the burning sensations on the surface, the absorption of imperfect matter, and the inordinate action of the heart. That I may not encroach too much, I will at present conclude by proposing the following theory of typhus fever:

Impaired energy and functional disorder of the nervous system, and, in consequence, prostration of strength, and imperfect secretion generally; irritation from the imperfect products of secretion, and, in consequence, increased nervous energy and sense of heat; absorption of the imperfect products, and, in consequence, inordinate vascular action.

*Alnwick; August*, 1827.

---

*Pulsation of Veins.*—In our Number for August, we inserted a remarkable case of pulsation of the veins from the Dublin Hospital Reports: as bearing upon the theories of the circulation at present exciting so much discussion, we subjoin a short account of an analogous case, taken from the *Philadelphia Journal.*

In the month of October, 1821, a child of Mr. C. V. of this city, aged about thirteen years, was attacked with bilious fever. He was ill for a long time, but eventually his disease left him in a very debilitated state. He was remarkably emaciated,—so much so, that curiosity one day induced me to examine his skeleton-like arms; when, upon a closer view, I saw the superficial veins upon the back of his hands distinctly pulsate, synchronous with the beat of the heart and arteries. I observed this phenomenon for five successive days. It was less evident as the child advanced in convalescence, and, when he recovered his strength and flesh, the pulsation of the veins entirely ceased.

---

### PRACTICAL MEDICINE.

*Neuralgia.*—We have been favoured, by Mr. Hutchinson, of Southwell, with the following copy of a letter addressed to him, and which adds another to the many proofs he has furnished, of the efficacy of Carbonate of Iron in neuralgic affections:—

"My dear Sir,—In compliance with your request as to the particulars of my complaint, it may be proper to inform you, that, about fourteen years ago, I was attacked with excruciating pain, the seat of which appeared to be confined to the eye, cheek-bone, and temple of the right side. This pain came on regularly about six in the evening of every other day, and continued, with short intermissions, till about four in the morning, when it suddenly left me. Between these attacks, I at first felt perfectly well; but, in the course of five or six weeks, I became irritable, and experienced a feeling of debility which was altogether new to me, and at this time sleep so entirely forsook me that it was thought advisable to administer narcotics, of which I took a variety: they produced distressing effects, without alleviating my pain.

I was now prevailed on to try Brathwaite's black-drop, of which I took nine drops every night for six months; at the end of which time I had lost all symptoms of my complaint, and, on leaving off this medicine, which I did gradually, I slept as well without as with it.

Perhaps you ought to know that the first attack of this malady made its appearance immediately on the repose into which my mind sunk on the recovery of a friend, the progress of whose alarming illness I had watched night and day.

I had no return of my complaint for upwards of a year, but from that time was liable to occasional relapses till January 1826. About this time, being in London, I was attacked with violent pain in the muscles of my neck on the right side. Considering this to be nothing more than what is called a stiff neck, I neglected it, and left London in an open carriage, the latter end of January. After driving about an hour, the pain in my neck became so violent that it was with difficulty I could hold my whip, and it increased to the end of the stage; when, after sitting still for a few minutes, I became perfectly easy. The pain, however, invariably returned with exercise, and abated with rest as before, throughout my journey home, 130 miles. The day after my return, although I was completely at rest, my pain returned with increased violence, and I fomented the part with laurel-leaves and water, as hot as I could bear, twice a-day, for several weeks, deriving only temporary relief. A blister, which was now applied, had a decidedly bad effect, for the pain became more acute, and somewhat altered in its character, being more lancinating, and coming on at short intervals, whether in motion or at rest. Cupping was next resorted to, with no better effect. Pills of stramonium in a very trifling degree relieved me, but produced effects too distressing to admit of my continuing them. A plaster of extract of belladonna was worn for a considerable time, without any perceptible effect. Bark was next administered, in rather large doses, for about a fortnight, when I was under the necessity of discontinuing it. At this time the pain became more and more acute, returning at shorter intervals, shooting deep into my ear, and never failing to attack me with great violence when shaving.

I began now to take the Subcarbonate of Iron, (I believe a drachm three times a-day,) which I persevered in for about a month, without being sensible of any relief. In addition to my malady, I was attacked violently with rheumatism in my left hip and thigh, which was completely removed by twice using the vapour bath, in which remedy I was induced to persevere in the hope that it might reach my other complaint; in which hope I was disappointed.

I may here observe, that, previous to my illness, I had been in the constant habit of using strong exercise on foot as well as on horseback, feeling no fatigue whatever, after walking twenty or thirty miles, or even more, to dinner. By this time my health was so impaired, that my constitution seemed to be altogether giving way. I was advised to go to Buxton, where I tried the tepid and warm baths for three weeks, and returned home much as I went.

My medical attendant now advised me to consult you. Accordingly I did so on the 29th of August, 1826, when you immediately pronounced my malady to be tic douloureux: and here let me remark, that this was the decided opinion of all whom I had before consulted, both as to the attack in my neck and the complaint in my face, from which I suffered so severely for many years. I was much and, you may suppose, very agreeably surprised at the decided manner in which you unhesitatingly assured me that I should get well. It was no easy matter, however, to keep hope alive for the first two months, during which time I took your medicine without any visible effect, being still unable to walk even two miles without great fatigue. My sight, too, as well as my memory and hearing, had all failed me so greatly as to entirely shut out many sources of amusement and comfort; besides which, for many months I may safely say that I scarcely ever slept, for, even when free from pain, the general irritability of my system was such as entirely to preclude rest in any shape whatever. At this period my pain, as well as irritability, began slightly to abate, and I clearly perceived that my general health was improving; and, as time advanced, this improvement became more rapid, so that, during the last winter, I have been in the regular habit of reading every night for several hours by candle-night, which I had not been able to do for many previous years. My memory and hearing too greatly improved; and, as a proof of my increased bodily strength, you may recollect that, on the 15th of December, I called upon you after walking twenty-two miles on my way to Newark, which was eight miles farther, and this I did without any fatigue whatever.

I had all along continued to take your medicine, till about January 1827, when I felt myself so perfectly well that I discontinued it. About the latter end of February, feeling a slight return of pain, I, by your advice, took another packet of medicine with the former happy effect, and am now perfectly well.

Wishing you equal success with all your patients, I remain, dear Sir, very sincerely yours, W. H. WAYNE.

3, *Chandos-street, Cavendish-square; May* 21, 1827.

---

*Phagedenic Ulceration of the Gums in Children.*—We have received the following note from Mr. Bush, of Frome.

In the Journal for June, Mr. Thomson has given a paper on a species of phagedenic ulceration of the mouth, called "Gangrenous Excoriation, or Erosion," in which he recommends, and perhaps with much propriety, the Balsam of Peru. The disease is well known to practitioners, especially those resident in crowded manufacturing towns, as attacking the children of the lower orders, and more particularly such as are deficiently supplied with nutritious food, and have not the advantages of well-ventilated rooms, regular personal ablution, and frequent changes of clean linen. I have seen this disease to supervene on typhus, scarlatina, rubeola, and variola, and at all periods from one to ten years. It takes place in children of delicate constitutions, such as are of scrofulous diathesis. Having witnessed a great deal of difficulty in curing these cases by bark, wine, and the ordinary routine of practice, I was induced (some ten or fourteen years ago) to give the Carbon, and with the most happy results. Indeed, where the plan has been tried early, I do not remember an unsuccessful instance. The following is the formula I have commonly used; but, when the bowels have been too relaxed, I have formed the linctus with Syrup. Pap. Alb.—

℞. Carbonis Ligni, Mellis, āā ℥j. M. sumatur cochleare minimum sœpe, vel quartâ quâque horâ.

This serves as a local and constitutional remedy. I have used it very extensively in cases of ill-conditioned ulcers, accompanied by constitutional debility, both internally and as a local application, with marked advantage.

It is worthy of observation, that animal and vegetable oils, sugar, gum, starch, and other substances found to contain great quantities of carbon, are those which by experience are found to recruit and restore most efficiently debilitated constitutions.

*Frome; August* 16, 1827.

*Case of Intermittent in a Child of six Weeks, cured by Arsenic;* by Dr. Dewees.—On the 3d December, 1820, I was called to visit the child of Mr. J. P. aged six weeks. About two weeks before I saw it, it was attacked with a tertian, which, after a few paroxysms, changed into a quotidian. The chill took place about ten o'clock A.M. and would continue nearly two hours, fever would succeed, which was followed by sweat. During the chill, the child was very ill, lay almost motionless, and refused every thing. Bowels regular; a little sickness of stomach occasionally. I prescribed arsenic in the following manner:—

℞. Sol. Mineral Fowl. ʒj.; Aq. font. ℥xij. Six drops to be given every four hours.

As soon as there was any evidence of the chill coming on, three drops of laudanum were ordered to be administered.

On the third day after commencing with the arsenic, the duration of the chill was much diminished, and the intensity of the fever abated; and in four days more entirely left it. The little patient's bowels were occasionally opened by castor-oil.

The mother of the child, who resided during the summer and fall at Bush-hill, was severely affected with fever and ague up to the time of her labour, at which time it ceased. Shall we consider this a case in which the remote cause was conveyed by the mother during gestation? or shall we merely look upon it as an extraordinary case happening to so young a subject, and at a season of the year when this disease is of very rare occurrence? (*Ibid.*)

---

*Lepra cured by Fumigations.*—We have received the following note from Mr. Green, of Great Marlborough-street:—

Leprous cases being generally considered obstinate under any mode of treatment, you will perhaps give insertion to the following case in your Journal, as I am of opinion it will go far to show the utility of fumigations in those diseases. The following statement was sent to me by the young lady's medical attendant, Mr. Moore, and by which it will appear that the disease had existed upwards of nine years; and the patient's written detail of her own case, in my possession, shows the disease to have been of much longer continuance.

"*Belfast; 19th April,* 1827.

"In April, 1818, when I first saw Miss R. N., she had a spot, hardly the size of a farthing, on the back of the neck ; for which she took a few powders, Submur. Hydr. gr. j. om. nocte ; a little Ungt. Hydr. Nitr. was applied to the part. She had been affected with it in a slight degree two or three years previous to this time, but the above-mentioned remedies had removed it.

"In November, she had pills of Submur. Hyd., Tart. Antim., and Gum Guaiac. ; and at the same time applied an ointment of Mur. Hydr. and Axung.

"In spring, 1819, the complaint increased, and the treatment was much the same as last year. She improved in the summer.

"In May, 1820, powders of Submur. Hydr., Sulph. Aurat. Antim. and Pulv. Rhei, given occasionally; and Lot. Mur. Hyd. in Aqua Calcis, was used frequently.

"In August, Lac Sulph. and Borax were made into a paste with distilled vinegar, and applied over the whole head, previously shaved. The application was renewed every fortnight, and continued for three months, without any benefit. An ointment of Coculus Indicus, with Axunge, was tried a few times also, without any effect.

"In December, the Solutio Arsenicalis was tried, with some good effect, and was continued with occasional intermissions for four or five months: the dose was increased from four to about seven drops. An ointment of Arsenic with Axunge was also used, and continued a few weeks.

"In 1821, a bath was tried two or three times a-week, with three-quarters of an ounce of Sulphuret of Potass thrown into it, continued for four or five months; Decoction of Sarsaparilla, and Arsenical Solution; Arsenical and other ointments.

"1822.—Baths occasionally, with Sulphuret of Potass. In the summer, warm salt-water baths were useful. Different ointments.

"1823.—Baths acidulated with Muriatic Acid; Lotion Spt. Vini and Mur. Hydr.; Antimonial Wine, twelve drops three times a-day.

"1824.—An electuary of Lac Sulphuris, Antim. Tartar. and Conser. Rosæ. Solution of Mur. Hyd. applied to the spots. The head slightly rubbed with olive-oil at night. Arsenical Solution, commencing with four drops a dose, and carried as far as thirteen, which produced some head-ache.

"1825.—Continued the Arsenical Solution, and Muriatic Acid in the bath. In September, pills, Sulphuret. Antim. Aurat. and Ext. Cicut.

"1826.—Decoction of Sarsaparilla, Arsenical Solution, and Ungt. Sulphur. &c. During this summer, the warm weather and sea-bathing apparently removed the complaint; but during the winter it returned.

"It will be seen by the preceding, that a variety of remedies were used by Miss N—. The most efficient were the Arsenical Solution, and the Lotion of the Muriate of Mercury; the Decoction of Sarsaparilla also appeared to be useful. The state of the disease, however, was more influenced by the season than by any remedies. In the summer it was much improved, but during the winter and spring it became considerably worse."

I first saw this young lady on the 21st of May of this year, in company with Dr. M'Cabe and Mr. Gaskoin. The case was clearly one of Lepra Vulgaris, extending itself generally over the body and limbs. She was ordered the following medicines:—℞. Liquor. Arsenicalis gtt. iv.; Liquor Potassæ gtt. xv.; Aquæ fontan. ℥x.; Syrup. Papaver. ʒij. fiat haust. ter in die sumendus.—℞. Acid Acet. fort. (vulgo Acid Arom.) ʒj.; Aquæ fontan. ℥iij. fiant guttæ: this was to be applied to the spots morning and evening; and she was directed to commence the fumigations,—at first every other day.

In the course of a fortnight, the improvement was very evident: the spots on the forehead, temples, and about the ears, extending to the cheeks, had become of a dusky brown colour, which gradually became fainter; and, for ten days previously to her leaving off the fumigations, it was difficult to distinguish where they had been situated. This lady had no occasion to interrupt the plan laid down for her, and took in all but thirty-eight baths. She was one of those subjects that could bear the administration of arsenic without the distressing symptoms frequently consequent on the use of that medicine. The dose in this case was attempted to be increased to six drops three times a-day, but it occasioned some disturbance of the functions, and was therefore not persevered in. The application, too, I have frequently known to be of much use in similar cases; but, as all these remedies had been, under various forms, repeatedly tried previously to the conjoint use of the fumigations, without advantage, I think to the latter much is to be attributed in the cure, which has taken place in less than six weeks, although this lady had come to London, and prepared to stay twelve months for that purpose. The result was very gratifying to the patient and her friends, particularly when it is considered that she had for years been prevented from appearing in society, or entering into the usual amusements of young ladies, being obliged to cover her face and arms.

It only remains for me to state that I have seen numerous cases, similar in their nature, which did not yield with so much facility. Whilst Miss N— was under progress of cure, I had another patient sent to me by Mr. Brodie, the daughter of a medical gentleman, similarly affected, and here too the disease had existed about the same number of years. She began the baths a few days after Miss N—, but the progress was slow when compared with the case detailed: she is now, however, rapidly improving, and I think will be well in three weeks.

*Great Marlborough-street; July 12th,* 1827.

### SURGERY.

*Case of Cynanche Laryngea, in which the Operation of Bronchotomy was successfully performed.*—Dr. Cullen, of Edinburgh, gives the following account of a case of this kind, in the last Number of the *Edinburgh Med. and Surg. Journal.*

On the morning of the 14th of March, about five o'clock, I was requested to see the subject of the following observation, Mary Bone, aged thirty-five, residing on the Castle Hill. I found her labouring under a very severe affection of the breathing, attended with long, and difficult, and rare inspiration, during which time the muscles of the neck and the alæ nasi were called into action, and the blood was evidently sucked forcibly out of the jugular veins; expiration was short, but otherwise easy; the face was very much flushed, and bathed in perspiration; the eyes prominent and red; and the whole countenance expressive of the utmost anxiety. She had occasional paroxysms of coughing without expectoration; the pulse was at 120, full and soft; the skin moist; the voice uttered in whispers only. She pointed to the larynx as the seat of severe pain, and mentioned that she felt as if a heavy weight had been laid upon her breast-bone. On percussion, the whole of the chest sounded well; by the stethoscope, respiration was very faintly heard, but without any râle.

The patient reported that she had had a bad cold since the month of November, with cough, difficult breathing, and expectoration; the voice, she said, she had gradually lost; she could speak very little in cold or damp weather, but more easily on fine, clear, warm days. All the symptoms were aggravated during the night. She had not been under proper medical treatment. A few days before my visit, she partook plentifully of a mixture of black beer and whisky, with a view to promote sweating; but this evacuation did not take place, and her symptoms were much aggravated by the dose.

On comparing the symptoms with the history of the case, I was quite convinced that the patient laboured under cynanche laryngea in the chronic form, with great obstruction in the rima glottidis, threatening suffocation. The texture of the lungs, and the minute branches of the bronchi, I imagined to be sound, or nearly so, in consequence of the sonorousness of the chest, and perfectly natural sound of the breathing, on listening with the stethoscope.

As the state of the symptoms did not indicate bleeding, and the history of the patient and her strength did not seem to admit of it, I contented myself with giving her a brisk purgative of compound powder of jalap, followed at the end of an hour by a draught of opium and ether. A large blister was applied to the sternum, and the feet were bathed in warm water.

At half-past ten A.M. there was no improvement in the symptoms; the antispasmodic draught had given slight, but only temporary, relief; and the woman, now for the first time, gave me reason to fear spasm of the glottis, by informing me that within the last fortnight she was occasionally quite easy, with natural respiration, while sometimes her breathing became suddenly exceedingly laborious, and continued so for half an hour or an hour at a time.

At one P.M. the purgative had operated well; the blister was beginning to rise. Not only, however, was there no amendment, but the symptoms were even worse than before. The breathing, inspiration chiefly, was very slow and laborious, attended with alternate rising and emptying of the jugular veins; the cough was frequent, still without expectoration; and she now complained of acute pain of the right side, increased on pressure between the intercostal spaces, or by full inspiration. The pulse was 130, small and weak; the countenance flushed; skin hot and dry; look anxious: indeed, she seemed on the point of suffocation. Believing her in the utmost and immediate danger, and that there was no time to wait the result of further medical treatment, and deeming bronchotomy to be the only chance of safety left, with the approbation and assistance of my friend Dr. Craigie, I proceeded immediately to the operation.

An incision, two inches and a half in length, beginning about the middle of the thyroid cartilage, and terminating over the isthmus of the thyroid gland, was made exactly on the median line of the neck, through the skin and superficial fascia. Over the ligament that unites the cricoid and thyroid cartilages anteriorly, I readily found the cellular interspace which separates the muscles of the right from those on the left side; between which I dissected down to the crico-thyroid membrane. The latter was cut longitudinally; but, as the incision was too small to admit the large curved tube generally employed by Mr. Liston and others, I enlarged the wound to a proper size by slitting up the lower third of the thyroid cartilage, and then succeeded in introducing the tube. In the operation an artery of considerable size was divided; it was not large enough, however, to admit of a ligature. As soon as the larynx was opened, at each inspiration blood from this vessel was sucked in by the wound; but luckily it did not annoy the patient, and I was not forced to employ any artificial means to stop it. On the first introduction of the tube, a fit of coughing supervened, attended, to my great surprise, with expectoration through the canula of a tough semitransparent mucus, slightly tinged with blood. The expectoration was not free, and the matter could hardly be expelled, so that it was necessary to extract it fiom the tube by forceps, and other similar means. The cough soon subsided in a great measure, and then we had an opportunity of observing that the respiration was much easier than before; and the woman said she felt very much relieved.

Ten P.M.—The tube has remained quite steady in its situation, undisturbed by the motion or coughing of the patient. Her state is highly satisfactory; respiration is quite free; cough not frequent, and attended with expectoration as before; pulse 110, full but soft; countenance of good expression, not flushed; heat of surface moderate; complains chiefly of want of sleep.—An anodyne draught, with forty drops of the sedative solution of opium, was advised to be taken immediately; and a drachm of compound powder of jalap was directed to be given in the morning.

15th, ten A.M.—Has passed a good night, with tranquil and refreshing sleep; cough, with expectoration, continues, but the breathing is free; pulse 100, of good strength. Powder has operated well.—A pectoral mixture, with syrup of squill, to be taken occasionally.

Eight P.M.—Cough rather more troublesome; other symptoms as before; pulse rather feeble, with disposition to sweat.—Ordered to have occasionally a teaspoonful of wine and water; the anodyne draught and purgative to be taken as before.

16th.—Another good night; respiration continues easy. She has been troubled this morning with sickness and occasional vomiting; and complains of pain of right side, increased on full inspiration or pressure.—The wine to be stopped; the cough mixture to be continued.

Nine P.M.—Nausea and vomiting have entirely ceased, but the pain of side is aggravated. The right side, at its lower part, sounds rather dull on percussion, and the breathing can hardly be heard with the stethoscope; cough occasionally severe; expectoration very viscid, and can with difficulty be removed from the tube; pulse 100, full; heat of surface considerable.—To have a dozen leeches to the side, and a poultice applied; anodyne draught and purgative to be repeated; squill mixture to be stopped.

17th.—Leeches bled well, and pain of side greatly relieved; respiration free; cough moderate.

For the next week the symptoms continued to improve. The respiration gradually became more free, the cough less frequent, and the expectoration more and more transparent, and so viscid, that the tube sometimes became nearly obstructed by it. The pain of the side gradually abated; the sonorousness of the chest returned; respiration was again heard; and the pulse sunk to its natural standard. A moderate quantity of food was allowed; the patient gradually regained her strength, and described herself as free of complaint of any kind. The presence of the tube gave no annoyance or pain. The edges of the incision granulated, and yielded a natural, purulent, and moderate discharge.

With regard to the voice, I may remark that, after the operation, she could not make herself understood as long as the tube was left open: when she placed her finger on the mouth of it, she was then able to speak distinctly, but in whispers only.

The patient remained in the state above described till about the 28th of the month, a full fortnight after the operation, when the voice having become stronger, and no cough or difficulty of breathing remaining, I withdrew the tube. The wound kept open, and the patient breathed through it with great ease and tranquillity. In a day or two afterwards I brought the edges together, and coverèd the whole with adhesive strap and bandage, so that the patient now had the use of the larynx restored. This stoppage of the wound, and restoration of the natural passage, were very well borne, and it then became my object to heal the opening as quickly as possible by the application of suitable dressings. In a short time the whole wound was filled with granulations, but no contraction of them took place, so that there existed during a certain period a line or fissure as it were, through which the probe could easily be introduced into the larynx. This fissure, however, did not serve for the introduction of air from without; and it was only during a fit of coughing or in a hurried attempt to speak, that a small portion of air escaped from the trachea, with a distinct crepitus, indicative of the difficulty it had to pass.

By the application of the sulphate of copper twice or thrice, the contraction of the granulations was completed, and the wound was perfectly healed on the 20th of April, about seven weeks from the date of the operation.

On reflecting on the history of the above case, I am quite satisfied that the operation was called for by the state of the disease, and that I had a right to expect a successful issue from the affection being confined to the larynx. I was inclined to think the absence of expectoration a very favourable symptom, for the following reason:—In several of the cases in which I have seen bronchotomy performed, there was previously a copious expectoration, which immediately after became difficult, and sometimes entirely suppressed, in spite of great efforts to cough on the part of the patient. But, indeed, as soon as an opening is made into the trachea, the patient can no longer cough, and of course he can hardly expectorate. Coughing is a compound action: the first part of it consists of a full inspiration, followed by the strong action of the constrictor muscles of the glottis, which the expiatory muscles attempt to force. The second part consists of the sudden relaxation of the muscles of the glottis coinciding with a violent expiration, during which, and by means of which, mucus, pus, or other matters in the windpipe and *bronchi* are brought up by coughing. But, as soon as an opening has been made below the *rima glottidis*, the action of the constrictor muscles cannot take place, and therefore the expiratory muscles cannot acquire sufficient force by acting against their antagonists; so that, in such circumstances, the patient cannot cough,—he can only expire, and produce a current of air too weak for the purposes of expectoration. Hence it is that expectoration becomes sometimes so difficult after bronchotomy; hence it is that the entrance of a small quantity of blood is sometimes fatal. The patient cannot get rid of the mucus or blood, because he cannot cough; he can only expire. I remember to have seen bronchotomy performed on a stout and vigorous young man, threatened with immediate suffocation from an affection of the larynx. Previous to the operation he could expectorate, though with some difficulty; but still he brought up a sufficient quantity. As soon as the opening was made, the expectoration became much more laborious; the sputa were brought up by expiration, and that with so little force as to be hardly driven through the canula. In a few minutes he died suffocated; and after death we found, filling the trachea for about an inch, a small quantity of coagulated blood, which, in its fluid state, should have been easily expelled, had the patient been able to cough.

Hence it is that I consider the absence of expectoration a favourable symptom, both because there will be no risk of suppressing its expulsion by the opening, and because it indicates the absence of inflammation of the bronchi. In the case of Mrs. Bone, expectoration occurred immediately after the operation; which, I conceive, depended on the glottis being so far contracted, that a quantity of air sufficient for a forcible expiration could not be taken in; but, when the canula was introduced, then the inspiratory part of the process became complete, and the expiratory difficult on account of the want of the glottis, the invariable effect of the new opening below.

On the whole, then, I have reason to be satisfied with the issue of this case, and recommend it to the notice of the Society, as illustrating some points of importance in deciding the question as to operating.

*Case of Cynanche Laryngea, in which the Operation of Tracheotomy was performed.* On the 10th of March, 1825, (says Mr. WHITE, in the *Dublin Hospital Reports,*) I was called to see John Duff, cabinet-maker, thirty-three years of age, a thin delicate looking man. He was sitting up in bed, bent forwards, and labouring under the following symptoms:—His breathing was hard and hissing; he had constant fits of suffocative coughing, and expectorated with difficulty some mucus. Pulse 130; tongue foul: he referred to the region of the larynx as the principal seat of his uneasiness; he could not bear much pressure upon the part; there was no external swelling; the fauces were redder than natural, and slightly œdematous, and there was no appearance of ulceration, neither was deglutition impeded. The account which this patient gave was, that he had lately sustained a severe attack of fever, which was followed by hoarseness, accompanied with an hard dry cough and soreness of his throat; and, having incautiously exposed himself to cold, these symptoms gradually increased, when he became alarmed and sent for me. I ordered him to be bled from the arm, to take a strong purgative without delay, to inhale warm vapour, &c. and one of the following pills every fourth hour: ℞. Submuriatis Hydrargyri gr. xxiv.; Pulv. Antimonialis gr. xvi.; Pulv. Ipecac. gr. xij.; Conserv. Rosæ q. s. ft. pil. xij.

The next morning, 11th March, he did not appear better, having spent a very uneasy night, with frequent attacks of coughing and dyspnœa.—Medicines ordered to be continued.

On the 12th, eight o'clock A.M. he was evidently worse; so much so, that, if I could have obtained his consent to perform tracheotomy, I should have felt myself justified in proceeding without delay to the operation.—I ordered leeches to be applied to the larynx, a large blister over the sternum, and his pills to be continued.

13th.—All his symptoms were augmented, and his wife informed me (for he was now unable to speak) that she apprehended his being suffocated several times during the night. The mercury had touched his mouth already; so, under these circumstances, I gave up all hope of relief from further medical treatment, and, at eleven o'clock P.M., finding his situation so alarming, I proceeded to the operation, assisted by Messrs. H. Carmichael and T. Roney. Having made the external incision of sufficient length, and cut through the fascia, I came down on the muscles, and, on separating the edges of the sterno-thyroid muscle, the two thyroid veins were exposed, together with a considerable arterial branch, the pulsation of which was quite perceptible, directing its course upwards towards the cross slip of the thyroid gland. Having pressed aside the artery and one of the veins with my finger a little to the right, I then cleared away some cellular membrane, and laid bare the trachea. A sharp-pointed bistoury being introduced between two of its rings, I cut directly upwards about half an inch in length, when the air escaped with considerable force, and much muco-purulent matter was expelled. Finding a mere division of the part not sufficient to allow a free discharge of the accumulated fluid, which was of a viscid ropy nature, I cut off lateral slips of the cartilages which leaving ample space, the patient breathed with freedom and expectorated large quantities of mucus. I attempted to introduce a silver canula into the wound, but the irritation, from the necessity of continually withdrawing and replacing the instrument, in order to carry off the mucus rapidly collecting about the mouth of the aperture, at length compelled me to abandon the use of the tube. In fact, as the parts lay sufficiently open, I determined not to interfere any further, at least for the present. His improvement was now most evident, and it was gratifying to witness his sudden relief from impending suffocation. A careful pupil was left with him to wipe away the discharge which occasionally obstructed the inner opening, causing temporary distress and difficult breathing.

The patient had intervals of sleep, disturbed by occasional efforts to get up the tracheal fluid: in every other respect he was as well as our most sanguine wishes could have anticipated.

To give a minute detail of the different symptoms which occurred from day to day, would occupy too much space: I shall, therefore, confine myself to noticing one or two of the more distinguishing features of this successful case.

A severe ptyalism set in, which reduced him much. In about six days after the operation, the external wound began to granulate and close: I therefore cut away the granulations, and was enabled to introduce a leaden canula, which he now bore without any uneasiness, and from which he seemed to derive considerable relief. He made several attempts to breathe through the glottis, but to no effect, and, on the 14th of April, his former symptoms returned, and became so urgent that I was obliged to enlarge the opening. I had introduced an ivory canula of a conical shape, and slightly curved, which filled up the entire opening, but was only admitted so far as to lie in contact with the edges of the tracheal aperture. This instrument has answered so well that the patient wears it at present attached to a leathern stock, which he buckles behind his neck, and is sufficient to keep the tube in its proper situation. It is occasionally taken out, in order to wipe away the mucus which gathers about it.

It is now two years since the operation was performed, and the patient, whom I saw this day (March 17th), has during that time worn the tube without any inconvenience, or being in the least prevented from working at his trade. The sides of the opening, which is of an oval shape, and one inch in depth to the trachea, are perfectly healed, smooth, and covered with a thin cuticle. The rima glottidis still remains closed, yet not so much so but to yield to slight expiration, while he is incapable of performing the act of inspiration through the glottis; or of speaking, without closing the aperture of the canula with his finger. On examining the fauces, the epiglottis appears thickened and standing erect. The artery, which was met with in the operation, situated on the anterior surface of the trachea, can be plainly felt at the inferior part of the opening.

---

*Ligature of the Arteria Iliaca Communis, at its Origin.*—Professor Mott, of New York, (says the editor of the *Philadelphia Journal*,) has recently performed this operation for the cure of aneurism, which, although but of ten days' standing, occupied the whole extent of the vessel, from within the ligament of Poupart to some distance above the origin of the internal iliac artery. The tumor was of large size, protruding the belly considerably at the iliac region; the patient suffered excruciating pain, which appeared to increase as the tumor enlarged. Dr. Mott's incision extended from the external abdominal ring to one or two inches above the crest of the ilium, dividing the tendon of the external oblique, and cutting through part of the origins of the internal oblique and transversalis. He then cautiously raised the peritoneum with his fingers, and succeeded in detaching it entirely from the tumor and vessels, without doing it the slightest injury.

The artery was then examined, and the aneurismal dilatation was found to cease at about half the distance between the bifurcation of the aorta and the origin of the internal iliac branches. The ligature was passed from the outside of the vessel, by the aid of the excellent instrument recommended by Drs. Parish and Hewson, carefully avoiding the iliac vein. The protrusion of the intestines rendered this part of the operation the most difficult. After the ligature was passed around the vessel, the wound was held open in such a manner as to allow the medical gentlemen present to see, and satisfy themselves of the exact situation of the ligature, which was just below the bifurcation of the aorta into the primitive iliacs, and on the side of the sacro-vertebral promontory. The ligature was then drawn tight and secured, the pulsation of the tumor ceased, its size was much diminished, and the patient was relieved from the agonising pain, previously unremitting.

The wound was lightly dressed, and the patient put to bed: the limb of the side operated on was cold, as might be anticipated; it was wrapped in cotton, and covered up, to preserve the temperature, until the circulation should be restored. To the great surprise and gratification of the surgeon, in little more than an hour after the operation, the circulation and temperature were entirely restored, and all fear respecting the supply of blood to the limb effectually dissipated.

No untoward circumstance has since occurred, now nearly a month, subsequent to the ligature of the artery. The patient has complained of no inconvenience, but the peculiar sensation of fulness or tension in the limb, as if the small vessels had not yet become accustomed to their new office in sustaining the great mass of the circulation for the support of the member. We heartily rejoice at the successful result of this case, for the sake of our fellow creatures, the character of the profession, and the reputation of Professor Mott, who is not more distinguished for admirable skill in operating and the success of his practice, than for those qualities of head and heart which endear him to such as are under the necessity of submitting to the last and dreadful resort of surgery, the knife.

---

*Operation for the Cure of Abscess in the Chest.*—We had the satisfaction (says the writer last quoted) of witnessing a very interesting and successful operation a few weeks since, performed by Dr. Mott, in the presence of Drs. Vandeberg and Proudfoot, for the cure of an abscess of the chest. The patient was a very interesting girl, about sixteen, who had some time before suffered from an attack of pleurisy, which, for want of proper depletion, had been followed by the formation of matter in the chest. The sternum at length became affected, and caries ensued, forming an opening through this bone, near the attachment of the third rib, though rather above it, whence matter was discharged in variable quantities. The effects of this suppuration were becoming very serious, and the life of the girl must evidently have sunk under the malady. She was sent to New York for the purpose of being relieved by an operation, should it be deemed practicable to operate with advantage. A steel sound, when passed through the opening in the sternum, could be introduced obliquely downwards as far as an inch or more, posterior to the junction between the sixth rib and its cartilage, and, judging by the sensation, the inferior part of the sinus was directly against this rib. Dr. Mott then made an incision under the left breast for three or four inches, laying bare the rib, which then gave a more distinct intimation of the extremity of the abscess when the sound was employed. He then cut carefully along the upper edge of the rib, detaching it from the subjacent texture; but the pleura appeared to be altered, or a substance formed behind it, which, when cut, seemed to have a gristly firmness. In order to secure the desired result, of obtaining a dependent opening to this abscess, he sawed out a triangular portion of the rib; then, cutting through the morbid texture above mentioned for about the fourth of an inch, he laid bare the extremity of the sound introduced through the sternum.

From this inferior opening the sound could be introduced nearly horizontally towards the spine, passing over the pericardium and heart. The greatest quantity of matter flowed from this part of the chest. Adhesion had apparently taken place between the pleura pulmonalis and costalis throughout the chest, beyond the limits of these sinuses. By this operation a beautiful and estimable young woman has been restored to health and society, who otherwise must have lingered for a time in hopeless anguish, and sunk into an untimely grave.

---

*Operation for Re-establishment of the Urethra.*—Dr. David L. Rogers, of New York, has recently operated with complete success upon a patient in the following condition:—In consequence of a prolonged gonorrhœa, a gentleman, aged forty-five, was afflicted with numerous strictures, which at length entirely prevented the flow of urine, and rendered it impossible to introduce a catheter. Dr. R. made an incision through the perineum, two inches in length, down to the bulb of the urethra, which was found to be very much contracted, and from the posterior part of the bulb throughout the whole of the membranous portion to the prostate gland, the urethra was so much changed in character as to resemble a small cord, its cavity being too small to allow of the introduction even of the finest probe. A straight sound was then passed through the anterior part of the urethra, and the strictures above the bulb forced; the end of the staff was then cut upon, the remainder of the tube being impervious, as before mentioned. A puncture was made into the urethra near the prostate, for the evacuation of the urine. The portion of the urethra between the end of the staff and this puncture, about two inches in length, was laid open, and a catheter passed into the bladder. The edges of the wound were then brought together over the catheter, and secured. The catheter was kept in the bladder for three weeks; it was withdrawn on the 6th of April: the patient continues entirely well, and to all appearance the operation has been completely successful. (*Philadelphia Journal.*)

